# NFATc1/αA: The other Face of NFAT Factors in Lymphocytes

**DOI:** 10.1186/1478-811X-10-16

**Published:** 2012-07-05

**Authors:** Edgar Serfling, Andris Avots, Stefan Klein-Hessling, Ronald Rudolf, Martin Vaeth, Friederike Berberich-Siebelt

**Affiliations:** 1 Department of Molecular Pathology, Institute of Pathology, University of Würzburg, Josef-Schneider-Str 2, D-97080, Würzburg, Germany

**Keywords:** Activation induced cell death/AICD, Anergy, Apoptosis, Calcineurin, NFATc, NFATc1/αA, NF-κB, Proliferation

## Abstract

In effector T and B cells immune receptor signals induce within minutes a rise of intracellular Ca^++^, the activation of the phosphatase calcineurin and the translocation of NFAT transcription factors from cytosol to nucleus. In addition to this first wave of NFAT activation, in a second step the occurrence of NFATc1/αA, a short isoform of NFATc1, is strongly induced. Upon primary stimulation of lymphocytes the induction of NFATc1/αA takes place during the G1 phase of cell cycle. Due to an auto-regulatory feedback circuit high levels of NFATc1/αA are kept constant during persistent immune receptor stimulation. Contrary to NFATc2 and further NFATc proteins which dampen lymphocyte proliferation, induce anergy and enhance activation induced cell death (AICD), NFATc1/αA supports antigen-mediated proliferation and protects lymphocytes against rapid AICD. Whereas high concentrations of NFATc1/αA can also lead to apoptosis, in collaboration with NF-κB-inducing co-stimulatory signals they support the survival of mature lymphocytes in late phases after their activation. However, if dysregulated, NFATc1/αA appears to contribute to lymphoma genesis and – as we assume – to further disorders of the lymphoid system. While the molecular details of NFATc1/αA action and its contribution to lymphoid disorders have to be investigated, NFATc1/αA differs in its generation and function markedly from all the other NFAT proteins which are expressed in lymphoid cells. Therefore, it represents a prime target for causal therapies of immune disorders in future.

## NFAT Factors^a^

The five members of the family of NFAT transcription factors are characterized by an evolutionary conserved DNA binding domain of approximately 300 amino acid (aa) residues. This domain forms a three dimensional structure similar to the DNA binding domain of Rel/NF-κB factors and, therefore, was designated as Rel Similarity Domain (RSD) or Rel Homology Domain (RHD)[1-3]. While the most distantly related NFAT5 shares 41-45% sequence similarity in its RSD with those of genuine NFATc factors NFATc1, c2, c3 and c4, the latter exhibit 68-75% sequence homology between their RSDs [4]. The DNA core motif for the DNA binding of NFAT factors, A/T GGAAA, resembles one half-site of κB motif of NF-κB factors, and there is a subset of κB binding motifs to which NFAT factors can also bind [5,6]. This was shown at the molecular level for the binding of NFATc2 homo-dimers to the κB motifs within the HIV LTR and the *Il8* promoter which share the core motif of NFAT binding sites [7,8]. Since NFAT and NF-κB proteins contact identical nucleotides, it is unlikely that both factors can bind at the same time to the same motif. Instead, they appear to compete for DNA binding. This seems to depend on the cellular concentration of both types of factors and other parameters, such as the level of co-factors, as AP-1, which affects the binding of NFATs to DNA.

Due to the control by the Ca^++^/calmodulin-dependent Ser/Thr-protein phosphatase calcineurin (CN; also known as PP2B) [1-3], the four genuine NFATc proteins constitute an own family of transcription factors with individual properties. Although they were described first for human Jurkat leukemic T cells [9] and murine EL-4 thymoma cells [10], they are not only expressed in lymphocytes but also in numerous other cells of the hematopoietic system, and also in cardiomyocytes, muscle, bone and brain cells. While in numerous studies the function of NFAT factors has been investigated in these tissues, in greatest detail the expression and function of individual NFATc proteins (including their isoforms) has been analysed in lymphoid cells [1-3].

In lymphocytes the three NFATc proteins NFATc1 (also known as NFAT2 or NFATc), c2 (NFAT1 or NFATp) and c3 (NFAT4 or NFATx) are expressed. Inactivation of individual *Nfatc* genes in mice revealed first indications for a functional dichotomy between the three proteins. Whereas due to defects in differentiation of embryonic heart, inactivation of the *Nfatc1* gene led to the early death of mouse embryos around day 14/15 upon gestation [11,12], *Nfatc2*^*−/−*^ mice were born at the normal Mendelian ratio and did not show any abnormalities within the first weeks upon birth [13-15]. However, with age *Nfatc2*^*−/−*^ mice developed a hyper proliferative syndrome and elevated primary and secondary immune responses. Due to delayed apoptosis, *Nfatc2*^*−/−*^ mice of 6 months and older harbour at least twofold more peripheral lymphocytes suggesting a suppressive role for NFATc2 in the generation of effector lymphocytes [13,14,16]. Several properties of *Nfatc2*^*−/−*^ mice were found to be markedly enhanced in mice double-deficient for NFATc2 and c3 [17]. Those mice showed a hyper proliferation of peripheral T and B cells leading to massive splenomegaly, a tendency to generate Th2 cells and, thereby, an enormous increase in production of Th2-type lymphokines [18]. In addition, lymphocytes double-deficient for NFATc2 and c3 show a strong decrease in Fas ligand expression and resistance to apoptosis [17,19]. *Nfatc3*^*−/−*^ mice, on the other hand, showed a 50% reduction in number of peripheral T lymphocytes which might be due to an increase in apoptosis rate. On the other hand, no obvious defects were observed in lymphokine (IL-2, IL-4 and IFNγ) production, and a slight decrease was detected in generation of single positive (SP) CD4^+^ and CD8^+^ thymocytes [20]. When conditionally inactivated in early double-negative (DN) thymocytes (in *Nfatc3*^*fl/fl*^*+ lck-Cre* mice), obvious defects were detected at the level of DN3 and double-positive (DP) thymocytes stages implying a role for NFATc3 in β selection and positive selection of thymocytes [21].

Contrary to peripheral lymphocytes from NFATc2 and c3-deficient mice, *Nfatc1*^*−/−*^ lymphocytes from mice generated by complementation of *Rag*^*−/−*^ blastocytes with *Nfatc1*^*−/−*^ embryonic stem cells showed a marked decrease in proliferation upon immune receptor stimulation [22,23]. This became obvious upon challenging *Nfatc1*^*−/−*^ T cells with αCD3 (+αCD28), concanavalin A or PMA + ionomycin, while no difference in their IL-2 synthesis was detected [22]. In distinct contrast to NFATc2 + c3-double-deficient T cells, *Nfatc1*^*−/−*^ effector T cells produced markedly less – and not more - IL-4 (and IL-6) upon αCD3 stimulation than T cells from wild type mice [22,23], and αIgM stimulation revealed a decrease in BCR-mediated proliferation capacity of *Nfatc1*^*−/−*^ splenic B cells [22]. Similar results were observed upon conditional ablation of NFATc1 in B cells [24]. While in such *Nfatc1*^*fl/fl*^*+ mb1-Cre* (or *Cd23-Cre*) mice, splenic B cell development appeared to be normal, in agreement with earlier observations [25] a 5–10 fold reduction in peritoneal B1a cells was observed. In addition to their low proliferation rate upon BCR stimulation, *Nfatc1*^*−/−*^ splenic B cells showed a decrease in their capacity to stimulate T cells and an increase in AICD and IL-10 production. Moreover, a mild clinical course of Experimental Autoimmune Encephalomyelitis (EAE) was observed for *Nfatc1*^*fl/fl*^*+ mb1-Cre* mice. Several of these defects appeared to be due to the diminished Ca^++^ flux in *Nfatc1*^*−/−*^ B cells whose proliferation is controlled by persistent Ca^++^/CN signals [24].

## The Ca^++^/calcineurin-mediated induction of NFATs in lymphocytes

NFATc factors differ from most other transcription factors by their strict dependency on Ca^++^ signals and CN activity [26] which induce the nuclear translocation and, thereby, activation of NFATc proteins. In the cytosol of effector lymphocytes and of most T and B cell lines, NFATc proteins are heavily phosphorylated and appear to be in complexes with the non-coding RNA NRON (Nonconding [RNA] repressor of NFAT) [27]. NRON creates a platform for larger RNP complexes [28] containing, in addition to NFAT, the three NFAT kinases casein kinase 1 (CK1), glycogen synthase kinase 3 (GSK3) and dual specificity tyrosine phosphorylation regulated kinase (DYRK), and calmodulin with further proteins [29].

**Figure 1:**
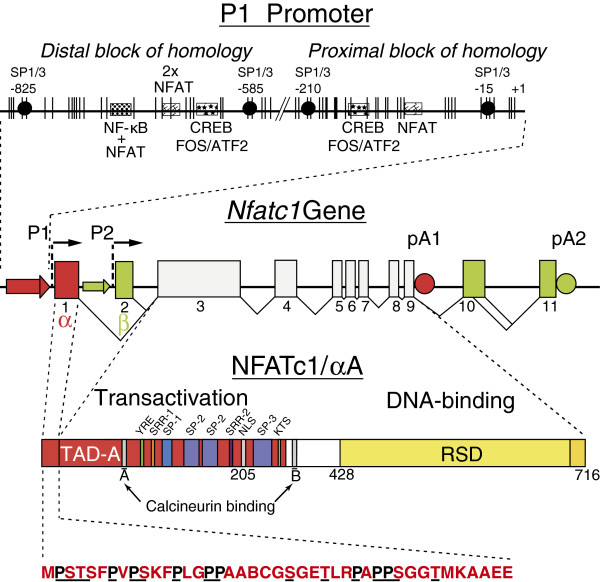
**Scheme of the mouse*Nfatc1*gene, its inducible promoter P1 (above) and the inducible short isoform NFATc1/αA (below) (modified according to refs.[**[35]**,**[44]**,**[49]**,**[50]**,**[96]**]).** In the promoter sequence, vertical dashes indicate CpG residues. Binding sites for transcription factors are shown as filled circles (for Sp1/Sp3 binding) or as boxes for the binding of various other factors. The murine *Nfatc1* gene spans approximately 110 kb DNA and is divided into 11 exons. Its expression is directed by two promoters, P1 and P2, and two poly A addition sites, pA1 and pA2. For the generation of NFATc1/αA RNA (encoding the α-peptide), the induction of P1 promoter results in the transcription of exon 1, splicing to exon 3 and poly A addition at the poly A site pA1. RNAs encoding the β-isoforms (with the β-peptide encoded by exon 2) are directed by promoter 2. The sequence of the N-terminal α–peptide from NFATc1/αA is given below. For NFATc1/αA, the Rel Similarity Domain, RSD, and transactivation domain, TAD-A, are indicated. In analogy to other NFATc factors (see Refs. [1-3]), two sites A and B for the binding of calcineurin, a nuclear localization signal, NLS, and several Ser-rich motifs that are phosphorylated, are also indicated. Below NFATc1/αA, the aa sequence of N-terminal α-peptide is shown in which all Pro and Ser/Thr residues are underlined.

Within minutes, immune receptor triggering leads to a rise in free intracellular Ca^++^ by releasing Ca^++^ from endoplasmic reticulum (ER) stores and, in particular, the influx of extracellular Ca^++^ through calcium-release-activated calcium (CRAC) channels. The details of this so-called store-operated calcium entry (SOCE) have been summarized in several comprehensive reviews to which we want to refer here [30-32]. Elevated free Ca^++^ level results in the binding of Ca^++^ to calmodulin followed by the binding of Ca^++^/calmodulin to CN which interacts through two “docking” motifs with NFATc proteins [33] (Figure 1). As a result of dephosphorylation of 13 aa residues within the regulatory region of NFATc2 [34], the nuclear localization signal of NFATc2 appears to be exposed followed by the binding of nuclear import factors [29] and rapid nuclear translocation. While the details of this process have yet to be elucidated, all three NFATc factors expressed in lymphocytes appear to be induced and translocated into the nucleus of lymphocytes in a similar way.

## The transcriptional induction and auto-regulation of NFATc1 in lymphocytes

In effector T and B lymphocytes immune receptor signals exert a secondary effect on NFAT activation, namely the strong induction of *Nfatc1* expression. Contrary to the *Nfatc2* gene which is constitutively expressed in peripheral effector lymphocytes, the transcription of the *Nfatc1* gene is strongly induced upon stimulation in peripheral T and B cells. This results in the predominant synthesis of the short NFATc1/αA isoform which spans 711 aa in mouse [35]. NFATc1/αA differs from the longer NFATc proteins by its short C-terminal peptide (Figure 1 and Table 1). Thereby, it lacks a second transactivation/repressor domain which has been described for the longer NFATc1 isoforms C [36] (spanning 925 [NFATc1/βC] and 939 aa [NFATc1/αC], respectively) and NFATc2 [37]. This domain harbours two highly conserved sumoylation motifs, and sumoylation of these motifs in NFATc1/C exerts a “dampening” effect on IL-2 production [38]. In addition, NFATc1/αA whose generation is directed by the inducible P1 promoter (Figure 1) differs from the NFATc1 β isoforms and NFATc2 by an unusual N-terminal peptide – the so-called α-peptide. The α-peptide spans 42 aa containing 9 Pro and 8 Ser + Thr residues in murine NFATc1/αA (Figure 1), compared to 1 Pro and 2 Ser + Thr residues in the 28 aa long N-terminal β peptide of NFATc1/β proteins. When expressed in EL-4 cells, NFATc1/αA shows a half-life between 2–4 h, whereas NFATc1/βA appears to be more stable, in particular upon stimulation of cells by TPA and ionomycin. And when fused to an estrogen receptor protein, the β-, but not the α-peptide is able to extend the half-life of the relatively unstable receptor protein in 293 HEK cells [39].

**Table 1:** Selected properties of individual murine NFATc proteins expressed in lymphoid cells

**NFATc Proteins**	**Length**^1)^	**Induction**^2)^	**SUMO-**	**Properties**	**Refs.**
	**[aa]**	**RNA/Protein**	**sites**^3)^		
NFATc1/αA	717	+/+++	(1)	anti-apoptotic, supports proliferation, anti-anergic, oncogenic activity	[24,38,39,90]
(NFATc,					
NFAT2)					
NFATc1/βA	703	-/-	(1)	n.d.	[38]
NFATc1/αC	939	+/+/-	2 (+1)	pro-apoptotic, (inhibits proliferation)	[35,38,39]
NFATc1/βC	925	-/-	2 (+1)	n.d.	[38]
NFATc2	923-927	(+)/-	2	pro-apoptotic, anti-proliferative, supports anergy induction, tumor suppressor activity	[24,35,56,57,59,90,97,98]
(NFAT1)					
NFATc3	1076	/-	1-2	(anti-proliferative)	[98]
(NFAT4,					
NFATx)					

1) Data from the NCBI database “Gene”. There, two short NFATc2 versions of 452 and 672 aa are also documented. However, their expression and function are unknown. For a comprehensive study about human and murine NFATc RNAs see Ref. [47].

2) Induction upon immune receptor stimulation in naïve and Th0/Th1 effector T cells and splenic B cells. RNA data were compiled from RNA Seq (E.S. et al., unpubl.) and real time PCR data obtained with splenic T and B cells upon primary stimulation [39]. The protein data reflect the results of Western blot assays [39]. +, 3–5 fold induction; +++, 50 fold induction and more.

3) Existence of SUMO-consensus sites within NFATc proteins. For NFATc1 and c2, their SUMOylation has been demonstrated *in vivo*[38,97]. (1) for NFATc1 indicates the existence of a “silent” SUMO consensus sites which remains un-used [38]. In NFATc3, two SUMO sites are present but only one corresponds to a “perfect” site.

NFATc1/αA is predominantly induced in Th0 and Th1 effector cells, whereas under conditions of T cell anergy, in regulatory T cells (Treg) and in Th9 and Th17 its induction is suppressed [39]. The suppression in Treg appears to be due to the binding of Foxp3, the cell lineage specification factor of Treg [40], to the inducible *Nfatc1* P1 promoter [41]. In conventional CD4^+^ T cells the induction of NFATc1/αA can be suppressed by ICER, an inhibitory short isoform of CREM, which is induced by high cAMP levels. Treg contain high levels of cAMP [42], and by direct contacts they elevate cAMP levels in conventional effector T cells. Thereby, ICER is translocated into the nuclei of effector T cells and binds to the two cAMP responsive elements (CRE) within the inducible *Nfatc1* P1 promoter [43].

In peripheral T and B cells, NFATc1/αA is the most prominent NFATc1 protein upon immune receptor stimulation. For the full induction of NFATc1/αA protein in primary T and B cells more than 24 h activation through immune receptors are necessary, whereas upon secondary stimulation of T cells, NFATc1/αA is fully induced within a few hours [24,36,44]. Triggering of co-stimulatory receptors, such as ICOS in T cells [45], enhances the immune receptor-mediated induction of NFATc1/αA. Since NFATc1/αA is also strongly induced in T cells double-deficient for NFATc2 and c3 [39], the induction (and maintenance?) of high NFATc1 levels does not seem to depend on NFATc factors other than NFATc1 (as on NFATc2, as previously reported [46]). The identification of *Rcan1* and *calcineurin Aβ/Ppp3cb* genes as NFATc1 targets in splenic B cells [24] suggests a regulatory circuit which guarantees persistent high expression of NFATc1/αA in peripheral lymphocytes upon antigen challenging by auto-regulation.

The murine and human *Nfatc1* genes span approximately 110 and 140 kb DNA, respectively, and are divided into 11 exons (Figure 1) (see also Ref. [47] for a detailed description of human and murine *NFATc* genes). The inducible transcription of NFATc1/αA is directed by the promoter P1 located in front of exon 1. P1 spans approximately 800 bp which are highly conserved between mouse and human. Similar to the promoter P2 located in front of exon 2, the P1 promoter corresponds to a CpG methylation island and forms a DNase I hypersensitive chromatin site in T cells [35]. In addition, the chromatin of the P1 promoter bears a number of epigenetic chromatin marks, such as H3K4me3 as a typical sign of active eukaryotic promoters [48]. In human Hodgkin´s lymphoma cell lines in which the *NFATC1* gene is suppressed all CpG residues of P1 were found to be fully methylated, whereas in Jurkat T cells they are de-methylated [49].

The P1 promoter can be divided into a proximal and distal block of homology, each spanning approximately 300 bp DNA. Both DNA blocks contain binding sites for NFAT factors, and in ChIP assays using EL-4 T cells it has been shown that they are bound by NFATs *in vivo*[39]. The NFAT binding sites within the distal block form a direct repeat of the NFAT core motif TGGAAA and bind NFAT factors with high affinity in EMSAs *in vitro*[35]. Upstream of these repeats, a composite NF-κB/NFAT motif is located to which NF-κB factors were bound in EMSAs, but which could also allow the binding of NFAT homo-dimers. In addition, the P1 promoter contains two CRE-like sequence motifs located in the proximal and distal block of homology. They share 100% sequence homology between mouse and man and were bound by CREB, Fos and ATF proteins in EMSAs [50]. The binding of CREB might be responsible for the poor transcriptional activity of the P1 promoter upon αCD3/CD28 or TPA and ionomycin stimulation in T cells and the stimulatory effect of cAMP signals on P1 activity which, on the other hand, do not exert a positive but suppressive effect on the induction of the chromosomal *Nfatc1* gene in T cells [35]. Whereas fusion of P1 to 5 kb upstream DNA did not result in a marked increase of its activity in transient transfections, fusion to a 1 kb DNA stretch from intron 1 of *Nfatc1* gene resulted in a 3–4 fold increase in P1 activity but did not change its inducibility [35]. However, when a DNase I-sensitive site from the last intron of the gene [51] was cloned downstream of a luciferase reporter gene whose expression was directed by P1, a 10–20 fold increase in P1-directed luciferase induction was detected (S. K.-H., unpubl. data).

In addition to P1 promoter activity, the generation of stable NFATc1/αA RNAs needs poly A addition at the proximal poly A site A1 near the end of exon 9 (Figure 1). For the human *NFATC1* gene, in transfection assays the pA1 site appeared as a “weaker” site than the distal *NFATC1* pA2 site or the proximal poly A site from the Ig μ heavy chain gene [44]. From these and further experiments we concluded that in naïve and resting peripheral lymphocytes in which the longer NFATc1 isoforms are predominantly formed the proximal pA1 site remains unrecognized resulting in the generation of long NFATc1 RNAs. Due to the massive increase in splice/polyadenylation factors in activated T cells [52] in these cells the pA1 site becomes bound by polyadenylation factors and, thereby, functionally active [44]. Surprisingly, the pA1 sites of *NFATc1* genes are not conserved between human and mouse leading to the synthesis of a 4.5 kb long NFATc1/αA RNA in mouse lymphocytes, compared to that of 3 kb in human T cells (Ref. [44] and unpubl. data).

## NFATc1/αA is not induced in anergic lymphocytes

The immune system does not only defend the organism against invading (micro-) organisms but also has to tolerate self-antigens to avoid auto immune diseases. In thymus, the majority of auto-reactive thymocytes are eliminated by the negative selection of DP thymocytes [53]. In addition to this central tolerance mechanism, in periphery the immune system is able to prevent unwanted immune reactions by rendering effector T and B cells unresponsive, or by eliminating auto-reactive lymphocytes. Several peripheral tolerance mechanisms can be distinguished, such as the generation of clonal anergy, adaptive tolerance [54], and the suppression of conventional CD4^+^T cells (and B cells) by Treg [55]. While peripheral tolerance mechanisms have been studied in numerous experimental settings [54], we will focus here on experiments in which NFAT factors have been investigated.

Treatment of T cells for 16 h or more with ionomycin diminishes their subsequent induction by TCR signalling without inducing apoptosis. This leads to a strongly reduced RNA synthesis of lymphokines IL-2, IFNγ, TNFα and GM-CSF upon secondary stimulation [56], a typical sign of T cell anergy. When the gene expression profiles of anergic Th1 cells (clone D5) were determined a dramatic change in gene expression was detected. By using DNA microarrays, approximately 5 fold less genes were identified than after (full) activation with PMA and ionomycin which had changed their expression 3 fold. Among those 200 genes about 1/3 was also detected in primary Th1 cells, including several genes encoding E3 ubiquitin ligases and proteases, such as Itch, Cbl-b and Grail [56,57]. Remarkably, in *Nfatc2*^*-/-*^ T cells which are more resistant to anergy induction than wild type T cells, the expression of approximately 35 genes – including E3 ligase genes – was reduced implying a specific role for NFATc2 in anergy induction. Under anergic conditions two prominent signalling proteins of TCR signalling, PLCγ1 and PKC-θ became ubiquitinated by Ca^++^ signals and degraded [57]. Those and further findings suggested that under ionomycin-mediated anergic conditions NFATc2 only - but not AP-1 (or NF-κB and, probably, further “stimulating” factors, such as NFATc1/αA) - is induced and binds to a subset of NFAT target genes, thereby stimulating the transcription of “anergy genes”. Within the promoters of *Rnf128* (encoding Grail) and caspase 3 genes (a further anergy-inducing gene) composite NF-κB/NFAT sites were detected to which NFATc2 homo-dimers were predominantly bound under anergic conditions. This led to the conclusion that the expression of a subset of anergy-inducing genes is controlled by NFATc2 homo-dimers [58]. NFATc2 was also shown to control B cell anergy (or adaptive tolerance) in MD4ML5 mice (expressing a HEL-specific BCR and transgenic HEL) [59] which are widely used to study B cell tolerance induction *in vivo*[60].

These findings suggest that NFATc factors in general exert a positive effect on E3 ubiquitin ligase expression and anergy induction [61]. However, published results and own experimental data suggest that NFATc1/αA plays an opposite role in anergy induction. In own experiments, the BCR-mediated induction of NFATc1/αA was found to be strongly impaired in anergic B cells from MD4ML5 mice (D.A.T. Pham, unpubl. results). When the *Rnf128* gene was inactivated in mice the expression of NFATc1 was found to be strongly enhanced upon αCD3 stimulation in naïve CD4^+^T cells and Treg [62], and in an oral tolerance model the nuclear appearance and, therefore, induction of NFATc1 was suppressed in CD4^+^T cells upon activation through their TCR and CD4 molecules [63]. Taken together, these and further data strongly suggest that contrary to NFATc2 (and, probably, the longer, non-induced NFATc1 isoforms) the inducible NFATc1/αA does not facilitate T and B cell anergy, but contributes to the optimal function of the immune system.

## NFATc1/αA induction is suppressed in regulatory T cells

Regulatory T cells (Treg) are generated in thymus as so-called naturally occurring regulatory T cells, nTreg, and in periphery as inducible regulatory T cells, iTreg. Both types of Treg are characterized by the expression of Foxp3, the constitutive expression of CD25 and the property to suppress the effector function of immune cells. Earlier work suggested an important role for Ca^++^ signals in the generation of nTreg [64]. It was also speculated that by binding near to NFAT sites, Foxp3 suppresses NFAT activity and lymphokine promoters [65].

This view found experimental support by studies on the interplay between Foxp2/3 and NFATc2. Determination of crystal structures of NFATc2-RSD and Foxp2-FKH (forkhead) domains formed with the distal NFAT site of the *Il2* promoter (the ARRE2 or Pu-b_d_ motif) suggested that Foxp3 could replace AP-1 in hetero-complexes formed with NFATc2 at the *Il2* promoter [66]. However, novel investigations on the interaction of FKH domain from Foxp3 with NFATc2´s RSD and Pu-b_d_ show that the structure of Foxp3´s FKH differs from that of FKH domain from Foxp2 [67]. These complexes consist of two RSD, four FKH domains and two DNA sites which were held together by one “domain swapped” FKH domain dimer. At Pu-b_d_, the FKH domain dimers contact in trans two different cognate Foxp3 binding sites which overlap. Thereby, the FKH domain of Foxp3 bridges two DNA molecules by binding to two individual Foxp3 sites in solution and brings them together. Concerning the interaction with NFATc2´s RSD, there is a larger interface between the RSD and Foxp3´s FKH domain than with that from Foxp2 suggesting that Foxp3 “fits better” to NFATc2 than Foxp2 [67].

While these structural analyses suggested that Foxp3 and NFATc2 could form heteromeric complexes in Treg and control their regulatory function (as proposed in Ref. [66]), they did not show that such complexes really exist in nTreg or iTreg *in vivo*. On the basis of X-ray crystallography, mutations were introduced into the FKH domain of Foxp3 which interfere with the interaction of FKH to the RSD of NFATc2 [66]. Infections of mouse CD4^+^ T cells with retroviruses expressing such mutant Foxp3 proteins led to conspicuous defects in Foxp3 and Treg function. All mutations within Foxp3´s FKH which impaired the interaction with NFATc2 also impaired the ability of Foxp3 to suppress *Il2* expression, and they impaired the expression of CD25, GITR, CD103, and, in particular, of CTLA4 which are all positively regulated by Foxp3 in Treg. Moreover, in a mouse model of autoimmune diabetes (the BDC2.5/NOD TCR tg mice [68]) co-transfer of CD4^+^ T cells expressing Foxp3 mutants were unable to suppress diabetes induction, whereas cells expressing wild type Foxp3 were able to suppress [66].

Earlier analyses of NFATc2 and c3-deficient mice indicated that the lack of both NFATc factors in mice did neither affect the generation of nTreg nor their function to suppress conventional CD4^+^ T cells. Instead, these assays suggested an intrinsic role for NFATc2 and c3 in CD4^+^ responder T cells to become suppressed by nTreg [69]. These data and those discussed above prompted us to investigate the role of NFATc1 in Treg by studying mice in which NFATc1 was conditionally deleted in T cells [70]. However, as in *Nfatc2*^*−/−*^*c3*^*−/−*^ T cells, no effect of NFATc1-deficiency was detected on nTreg formation and function in *Nfatc1*^*fl/fl*^*x Cd4-Cre* mice. The same was observed for T cells deficient for NFATc1 and c2, and again for T cells from mice double-deficient for NFATc2 and c3. However, when the generation of iTreg was investigated upon primary αCD3 + CD28 stimulation by incubation with TGFβ + IL-2 *in vitro* and *in vivo*, a reduction in iTreg formation was detected for T cells deficient for either NFATc1 or c2, and this reduction was strongly accelerated for T cells double-deficient for NFATc1 and c2, or NFATc2 and c3. Similarly, the generation of iTreg was strongly diminished when NFATc1/c2-double deficient T cells – instead of wild type T cells - were co-transferred into lymphopenic mice to induce colitis (which is an approach to induce iTreg under inflammatory conditions [71]). In ChIP assays an increase in NFATc1 binding was detected upon TGFβ treatment of CD4^+^ T cells to the intronic CNS1 enhancer element of the *Foxp3* gene whose activity was shown to be controlled by NFAT and Smad factors [72]. Foxp3 suppresses NFATc1 induction by binding to the P1 promoter and inhibits its activity [41]. In addition, Treg were shown to have a reduced Ca^++^ flux [73], CN activity and, thereby, NFATc activation [74].

## NFATc1 suppresses the Activation Induced Cell Death (AICD) of lymphocytes

Activation Induced Cell Death (AICD) is one route of apoptosis induction in lymphocytes which, when suppressed, contributes to cancerogenesis of lymphocytes and other cells [75]. NFATc1 and c2 exert contrary effects on AICD of peripheral B and T cells. Upon α-IgM stimulation murine splenic *Nfatc1*^*−/−*^ B cells show a higher apoptosis rate than wild type B cells, indicating an anti-apoptotic activity for NFATc1 and, particularly, for NFATc1/αA which is predominantly expressed under these conditions in splenic B cells [24]. Under the same conditions *Nfatc2*^*−/−*^ B cells were found to be resistant to apoptosis induction [24] supporting earlier work on the pro-apoptotic role of NFATc2 in BCR-mediated apoptosis in human Burkitt´s lymphoma B cells [76]. Similar results were observed for murine T cells indicating a dichotomy between NFATc1/αA and NFATc2 in apoptosis induction [35]. The expression of numerous genes which are known to affect apoptosis are regulated by NFATc factors in lymphocytes. Such as the promoters/enhancers of Fas ligand, CD40 ligand, PD-1, light and osteopontin genes [24,77-81], the promoter directing the expression of short isoform of caspase 8-inhibitor c-Flip in murine T cells [82] and several others. However, differences in NFATc1/αA or NFATc2 binding to the promoters/enhancers of these genes have not been observed. Therefore, it remains to be shown how the apparent contrasting effect of various NFATc proteins on AICD of lymphocytes is reflected at the molecular level. The strong induction of NFATc1/αA upon stimulation of T and B effector cells supports the speculation that high levels of NFATc1/αA can protect against AICD whereas low levels are unable to do so. However, when (naïve) splenic B cells in which large amounts of NFATc1/αA are generated upon BCR stimulation for 2 days *ex vivo*[24] are stimulated by α-IgM for 3 days and longer, they die by AICD, unless they are rescued by α-CD40, LPS or CpG which all induce NF-κB factors. This suggests that in immune responses NFATc1/αA and NF-κB factors collaborate in the clonal expansion (and differentiation) of lymphocytes. At the end of an immune reaction, the majority of lymphocytes will undergo apoptosis when they bear an excess of NFATc factors, whereas those with an “optimal” ratio between NFATc1/αA and NF-κBs will survive.

## NFATc1/αA as a putative oncogene which supports lymphomagenesis

Similar to the control of apoptosis, NFATc factors appear to exert dual functions in the generation of lymphoid and other malignancies. Genome-wide mappings of common insertion sites (CIS) for oncogenic retroviruses revealed a preference of insertions into the murine *Nfatc1* and *c3* genes. Whereas in total 24 provirus insertions were detected for the *Nfatc1* locus and 16 for the *Nfatc3* locus, only two insertions located more than 30 kb 3’ from the *Nfatc2* gene were detected, and no within or near the *Nfatc4* gene [83-88]. For the *Nfatc1* gene, all 24 proviral insertions were detected either around its promoter region or within (or very close to) intron 10, and a similar distribution of insertions sites was found for the *Nfatc3* gene. In hematopoietic cells, several DNase I hypersensitive sites, and in Th1 and Th2 cells the H3K4me3 chromatin mark, a sign for active transcription, were mapped within intron 10 of the *Nfatc1* gene [48,51]. These data suggest that oncogenic retroviruses insert into the transcriptionally active regions of *Nfatc1* gene to affect its expression.

The identification of common sites for retroviral integrations favours the *Nfatc1* and *Nfatc3* genes as putative oncogenes for the generation of lymphoid tumors. However, this does not exclude that the expression of human *NFATC1* gene is switched off in classical Hodgkin´s lymphoma cells in which BCR signalling is suppressed [49], and NFATc3 can function as a tumor-suppressor for retrovirus-induced T cell lymphomas [84]. Its pro-apoptotic activity favours, on the other hand, NFATc2 as a tumor suppressor, and it has been described as a suppressor of neoplastic changes in chondrogenesis [89].

Albeit not shown in lymphoid cells, the dual role of NFATc1/αA and NFATc2 in oncogenesis has been demonstrated by ectopic expression (of constitutively active [ca] versions of) both factors in NIH 3 T3 cells [90]. While caNFATc2 led to cell cycle arrest and apoptosis, over-expression of caNFATc1/αA led to an increase in proliferation and cell transformation. Moreover, lack of NFATc2 resulted in an elevated tumor formation induced by the tumor promoter methylcholanthrene. To a large part, the differences in oncogenic capacity between caNFATc1 and c2 could be attributed to the C-terminal peptide of NFATc2, since removal of the 230 aa long C-terminal peptide from NFATc2 (including its SUMO sites) released its tumor suppressor phenotype and “converted” NFATc2 to a NFATc1/αA-like protein [90].

There are several reports on the de-regulation of Ca^++^/CN-mediated induction of NFATc1 in human haematopoietic malignancies supporting the former finding on the “oncogenic nature” of NFATc1 (see refs. [91,92] for current reviews). For diffuse large B-cell lymphomas (DLBCL) [93] and pancreatic cancer cells [94] an NFATc1-directed up-regulation of the *MYC* gene has been described which appears to be due to the recruitment of histone acetylases to the *MYC* promoter [95]. This suggests that NFATc1 might also be involved in further human lymphomas in which c-Myc is over-expressed, such as in Burkitt´s lymphomas.

## Conclusions

The short NFATc1 protein NFATc1/αA differs in its structure and function from numerous, if not all other NFAT proteins. NFATc1/αA lacks a 250 aa residues long C-terminal peptide with two sumoylation motifs, it contains a specific N-terminal peptide with several Ser/Thr and Pro residues, and its synthesis is strongly enhanced in effector T and B cells by immune receptor signals. Several lines of evidence indicate that NFATc1/αA contributes – in collaboration with NF-κB factors - to the survival of effector lymphocytes. These properties favour NFATc1/αA as a target for treating disorders of the immune system.

## Endnotes

^a^For simplicity we use here and in all our other publications the term “NFAT factors” for members of a family of transcription factors which share a number of specific properties, e.g. the induction by calcineurin which de-phosphorylates an array of phosphorylated sites upon cell activation. We are aware that the term NFAT (Nuclear Factor of Activated T Cells) is misleading since these transcription factors are expressed in numerous other cell types as well, and the acronym NFAT indicates the term factor.

## Abbreviations

AICD, Activation Induced Cell Death; α-IgM, Antibody directed against the IgM B cell receptor; BCR, B Cell Receptor; cAMP, adenosine 3’:5’ cyclic mono-phosphate; ChIP, Chromatin Immunoprecipitation; CN, Calcineurin; CRE, cAMP Responsive Element; CREM, cAMP Response Modulator gene; CsA, Cyclosporin A; HEL, Hen Egg Lysozyme; ICER, the inducible cAMP early repressor; iTreg, Inducible regulatory T cells; NFAT, Nuclear factor of activated T cells; nTreg, Naturally occurring regulatory Tcells; PMA, Phorbol 12-myristate 13 acetate; TCR, T cell receptor; TPA, 12-O-tetradecanoyl phorbol-13-acetate.

## Competing interest

The authors declare that that they have no competing interests.

## Authors´ contribution

ES wrote the draft of the manuscript which was discussed in detail, modified and corrected by AA, S K-H, RR, MV and F B-S. Figure 1 was made by RR. All authors read and approved the final manuscript.

## Funding

This publication was funded by the German Research Foundation (DFG) and the University of Wuerzburg in the funding programme Open Access Publishing. 
